# A Systems Medicine Approach: Translating Emerging Science into Individualized Wellness

**DOI:** 10.1155/2017/1718957

**Published:** 2017-10-15

**Authors:** J. S. Bland, D. M. Minich, B. M. Eck

**Affiliations:** ^1^Personalized Lifestyle Medicine Institute, Seattle, WA, USA; ^2^Institute for Functional Medicine, Federal Way, WA, USA; ^3^University of Western States, Portland, OR, USA; ^4^Metagenics, Inc., Aliso Viejo, CA, USA

## Abstract

In today's aging society, more people are living with lifestyle-related noncommunicable diseases (NCDs) such as cardiovascular disease, type 2 diabetes, obesity, and cancer. Numerous opinion-leader organizations recommend lifestyle medicine as the first-line approach in NCD prevention and treatment. However, there is a strong need for a personalized approach as “one-size-fits-all” public health recommendations have been insufficient in addressing the interindividual differences in the diverse populations. Advancement in systems biology and the “omics” technologies has allowed comprehensive analysis of how complex biological systems are impacted upon external perturbations (e.g., nutrition and exercise), and therefore is gradually pushing personalized lifestyle medicine toward reality. Clinicians and healthcare practitioners have a unique opportunity in advocating lifestyle medicine because patients see them as a reliable source of advice. However, there are still numerous technical and logistic challenges to overcome before personal “big data” can be translated into actionable and clinically relevant solutions. Clinicians are also facing various issues prior to bringing personalized lifestyle medicine to their practice. Nevertheless, emerging ground-breaking research projects have given us a glimpse of how systems thinking and computational methods may lead to personalized health advice. It is important that all stakeholders work together to create the needed paradigm shift in healthcare before the rising epidemic of NCDs overwhelm the society, the economy, and the dated health system.

## 1. Chronic Disease and Lifestyle as Culprit

The world's population is aging. Although more people are living longer, it also means that more people are living with noncommunicable diseases (NCDs) such as type 2 diabetes, obesity, cardiovascular diseases, arthritis, chronic respiratory diseases, and cancers. The World Health Organization reported that NCDs have become a global health concern, as they currently accounted for 63% of annual global deaths [1], let alone the burden these chronic illnesses place on any healthcare system and the society.

NCDs progress very slowly, and most of them are linked to unhealthy lifestyle and behaviors. Four major unfavorable lifestyle choices—unhealthy diets, excessive use of alcohol, physical inactivity, and tobacco use—contribute significantly to the development of these NCDs, and these risky lifestyle choices often cooccur and therefore synergistically increase NCD risk and comorbidity [2]. It has been estimated that, by not smoking, being physically active, and adhering to a healthy dietary pattern, 80% of NCDs and premature death could be prevented [3].

The current predominant healthcare approach is essentially favoring “sick care,” a system that works well for treating acute diseases or acute worsening of NCDs. Clinicians and patients have come to expect immediate pharmaceutical solutions for instant cure or symptom relief. Not only is this concept inappropriate in treating NCDs, it largely ignores the subclinical stage of disease development and lacks ability and incentive to properly address disease-promoting lifestyles, the underlying causes of NCDs [4]. A drastically different approach to healthcare is in urgent need.

## 2. Personalized Lifestyle Medicine as First-Line Therapy

Acknowledging that the current healthcare system cannot efficiently accommodate the rising epidemic of NCDs, opinion-leader organizations have joined forces to advocate a new approach in NCD prevention and treatment: lifestyle medicine [2]. The American College of Preventive Medicine defines lifestyle medicine as “a scientific approach to decreasing disease risk and illness burden by utilizing lifestyle interventions such as nutrition, physical activity, stress reduction, rest, smoking cessation, and avoidance of alcohol abuse.” The American Heart Association (AHA), the American Diabetes Association (ADA), the American Cancer Society (ACS), the U.S. Department of Health and Human Services, and others, all have published population-based guidelines or recommendations for lifestyle medicine.

In reality, however, these “one-size-fits-all” public health recommendations (albeit some of which are stratified by age and sex) have been insufficient in meeting the need of our highly diverse human populations. For instance, consumption of foods low in glycemic index has been widely recommended for the management of hyperglycemia, a major concern in metabolic syndrome, diabetes, and obesity. Yet, several trials investigating the efficacy of diets low in glycemic index have reported mixed results due to a wide range of individual responses [5]. Others have found that, even as simple as consuming an identical portion of white bread or an identical meal, the postprandial glycemic response varied significantly from person to person [6–8]. Therefore, an effective dietary and lifestyle recommendation for glycemic control—and applicable to all other risk-reducing, health-promoting strategies—will need to take into account factors that cause interindividual differences.

## 3. The Case for Personalized Lifestyle Medicine

What makes each individual different is not just due to genetic variations. Environmental factors, epigenetics, gene-environmental interactions, and many others together modify our nutritional requirement, metabolism, and predisposition to disease, as well as our response to drug or lifestyle intervention. Integrating these pieces of information will allow healthcare professionals to provide personalized nutrition and lifestyle recommendations that minimize side effect and optimize efficacy. The application of systems biology to develop personalized healthcare is referred to as systems medicine.

Randomized controlled trials (RCTs) have been considered the gold standard of clinical research as they provide unbiased evidence of the average treatment effects in the intervention group(s) compared with the placebo group. On the other hand, in practicality RCTs can only investigate a limited number of factors at one time. By “cancelling out” the potential effects of other variables via randomization, RCTs reduce the complexity of real life and assume that all subjects in the intervention group would respond to the intervention more uniformly. Further, RCTs usually employ strict exclusion criteria resulting in a more homogenized study population and thus have limited generalizability to other types of population groups. Therefore, RCTs are impractical to provide complex evidence needed for individualized treatment.

This limitation is especially true in nutrition and lifestyle research. Foods do not behave like drugs, and nutrients are rarely consumed in isolation [9]. A diet or dietary pattern contains multiple bioactive food components and is difficult to eliminate the day-to-day variation in dietary intake [10]. The effects of food are more subtle and may take a significant amount of time to produce discernible results. Plus, differences in genetic makeup, metabolic variations, environmental exposure, and even variations in the gut microbiome all potentially affect how an individual absorbs, responds and utilizes the nutrients in a diet [11].

## 4. Harnessing OMICS Technologies in Personalized Lifestyle Medicine

Advancement in “omics” technologies is gradually pushing personalized lifestyle medicine toward reality. Researchers from the Vitamin D/Calcium Polyp Prevention trial found that the effect of vitamin D in the prevention of recurrent colorectal adenomas was significantly modified by a single-nucleotide polymorphism (SNP) in the vitamin D receptor gene [12]: vitamin D was beneficial in individuals with the rs7968585 AA genotype but harmful in those with 1 or 2 G alleles, indicating the importance of personalizing vitamin D supplementation. In the Preventing Overweight Using Novel Dietary Strategies (POUNDS LOST) trial, investigators reported that the fat mass- and obesity-associated (FTO) gene SNP modified appetite measures in adults with overweight. Specifically, those with the rs9939609 A allele benefitted the most from a high-protein weight-loss diet [13]. Another weight-loss trial in men with obesity found that baseline DNA methylation patterns in CpGs on the WT1 promoter might be used as epigenetic markers that predict outcomes in weight loss [14]. Other examples of research supporting personalized nutrition approaches have been summarized in our previous review [15].

In the context of personalized lifestyle medicine, however, the ability to analyze multiomics data is essential. High-throughput platforms such as genomics, epigenomics, transcriptomics, proteomics, and metabolomics have allowed powerful comprehensive analysis of how complex biological systems are impacted upon an external exposure, whether it is as simple as ingesting a single phytochemical compound or as complex as a new dietary and exercise regimen [16]. A seminal, proof-of-principle, work is the integrative personal omics profile (iPOP) study led by Dr. Snyder from Stanford University School of Medicine [17, 18]. This study collected multiomics data and autoantibody profiles from a single individual (Dr. Snyder himself) over a 14-month period. Assessment of these multiomics data over time revealed that the integrated information could identify a number of medical risks, predict the individual's health versus diseased states, and potentially monitor treatment responses. The researchers believe that iPOP would lead to better personalized healthcare by providing more precise methods of monitoring, targeted treatment, and prevention.

Through the technologies' ability to simultaneously evaluate complex gene and protein expression and the metabolic fingerprints, a healthcare professional can in theory establish a personal omics profile for each patient, taking into account the individual's unique genetic and epigenetic makeup, and record the progression of the omics data in response to intervention [17].

As more patients now believe the importance of healthy lifestyle in their health, clinicians and healthcare practitioners have a unique opportunity in advocating lifestyle medicine for NCD prevention and treatment because patients see them as a reliable source of advice [19]. In fact, patients' active participation is a crucial component in personalized lifestyle medicine [20]. Environmental data related to physical activity or nutrition may be collected via personal monitoring devices worn by patients. Studies have demonstrated that patients desire to be active participants in information exchange and the decision-making process, and those who are participatory yield better health outcomes than those who are not [21, 22]. With the integration of patients' personal omics profile into part of the routine clinical care, patients can be intimately involved in their health trajectory with the professional assistance of their healthcare providers.

## 5. Both Challenges and Promises Ahead

As promising as the omics technologies sound, translating personal “big data” into personalized, actionable, and clinically relevant solutions still faces various challenges and many aspects are still in progress. Among tens of thousands of newly discovered candidate biomarkers identified through omics research during the last decade, only a hundred or so biomarkers have demonstrated clinical utility [23]. There has not been a comprehensive genetic database that helps healthcare professionals predict how an individual's genetic makeup systemically affects nutritional requirements [24]. Information gathered beyond genetic testing, such as that from genomic, proteomic, and metabolomics platforms, increases exponentially in its complexity. Computational tools (e.g., MetaCore™, MetaboAnalyst, InCroMAP, and 3Omics) that are designed to integrate and interpret all the information are still in their infancy and facing various logistical challenges [25]. Further, there is a shortage in multiomics data that capture individuals' response to environment such as nutrition, physical activity, lifestyle, stress, and environmental toxins [26]. Machine-learning algorithms and new statistical methodologies that can integrate these multidimensional data are also needed.

Clinicians also face other practicality issues. What kind of training is required in order to deliver complex omics-based advice? How would all the sensitive genetic information of the patients be analyzed and stored? Are there programs or cloud-based services that can create a master tracking portal for all patient data? Would all the advanced testing and additional counseling be covered in the care payer systems? Are there reliable, mobile-based diagnostics and wearable technologies that can collect behavior and other health-related data? Are there nutritional products that can support personalized lifestyle medicine?

At the emotional level, although clinicians and health professionals have a key role in delivering personalized lifestyle medicine, the vast amount of information generated from the omics technologies can be overwhelming and technically challenging. Without simplified and clinically relevant recommendations, health professionals may find it difficult to deliver personalized care. Patients may be uncomfortable in disclosing detailed, intimate personal genetic information to clinicians, or they simply may not be motivated to be participatory. Also, patients and clinicians may have become accustomed to the current reactive, instead of proactive, approach to healthcare (“is there a magic pill to treat obesity?”). Whether these powerful new technologies and individualized recommendations can motivate patients in taking charge of their own health remains to be seen.

## 6. A Glimpse into the Future

Even though the road to personalized lifestyle medicine is challenging, emerging ground-breaking research projects have given us a glimpse of how systems thinking and computational methods may lead to personalized health advice.

A study conducted at the Weizmann Institute of Science (Rehovot, Israel) collected extensive phenotypic data—week-long monitoring of blood glucose, detailed recording of diet, and lifestyle information via smartphone technology, blood tests, anthropometrics, lifestyle and medical questionnaire, and stool samples for gut microbiome analysis—from 800 individuals. Through the machine-learning algorithm that the researchers developed, they were able to integrate all the gathered information and predict an individual's postprandial glucose response after a standardized meal [6]. This is an example of how big data are translated into personalized nutritional recommendations.

Another ongoing study, the Data-as-a-Service Platform for Healthy Lifestyle and Preventive Medicine (DAPHNE) project, aims to tackle obesity epidemic via personalized lifestyle medicine. By using mobile apps to continuously monitor lifestyle and behavior data in real time and a web-based health services portal, the project hopes to increase the individuals' awareness to their health trajectory and provide a holistic and individualized approach to treat obesity [27].

The Pioneer 100 Person Wellness Project (P100), a 10-month pilot project originated from the Institute for Systems Biology (Seattle, USA), aims to optimize wellness by integrating longitudinal information from whole genome sequencing, clinical and functional lab testing, gut microbiome analysis, and quantified self-measures from 108 individuals [28, 29]. The goal is to apply what the researchers learned to a much larger population and deliver early personalized healthcare to optimize health and prevent NCDs.

## 7. Conclusion

The current healthcare system is no longer sufficient in addressing NCD epidemic. Personalized lifestyle medicine will be the key to empower patients to regain control of their health. There are still logistic and technical challenges to overcome in order to deliver personalized advice based on systems thinking, molecular diagnostics, accurate environmental measurements, and advanced computational methods. Working groups such as the International Society of Nutrigenetics/Nutrigenomics and the AHA/ESC/EACPR/ACPM policy statement all aim to encourage integrated action by all stakeholders to make personalized lifestyle medicine a reality [2, 30, 31]. Healthcare professionals, geneticists, bioinformaticians, food industry, health industry, and policy makers will all need to work together to strengthen the science, to improve knowledge delivery, and to improve public education in order to create the needed paradigm shift in healthcare.
